# An Account of Diseases in an Eastern District of London, from April 20 to May 20, 1810

**Published:** 1810-07

**Authors:** 


### *An Account of Diseases in an Eastern District of London, from April 20 to May 20, 1810.*

| | |
|---|---|
| **ACUTE DISEASES.** | |
| Pneumonia | 4 |
| Peripneumonia Notha | 5 |
| Typhus Mitior | 4 |
| Rheumatismus Acutus | 3 |
| **CHRONIC DISEASES.** | |
| Tussis | 11 |
| Tussis cum Dyspnœa | 7 |
| Hæmoptysis | 4 |
| Phthisis Pulmonalis | 2 |
| Hydrothorax | 4 |
| Pleurodyne | 7 |
| Hepatitis Chronica | 3 |
| Hysterodynia | 4 |
| Fluor Albus | 7 |
| Dysuria | 5 |
| Obstipatio | 2 |
| Hemiplegia | 2 |
| Cephalalgia | 6 |
| Hernia | 2 |
| Rheumatismus Chronicus | 10 |
| **INFANTILE DISEASES.** | |
| Convulsio | 3 |
| Diarrhœa | 5 |
| Aphthæ | 4 |
| Scrophula | 6 |
| **PUERPERAL DISEASES.** | |
| Mastodynia | 4 |
| Stillicidium Urinæ | 1 |
| Menorrhagia Lochialis | 4 |
| Dolores Post Partum | 5 |

In the preceding list, several cases are referred to, of a disease which the Nosologists have denominated Hysteralgia. This is principally distinguished by pains in the loins, shooting forwards towards the region of the pubis.

Pains of this kind occur at different times, and under different circumstances. In some subjects they recur in a greater or less degree at every period of menstruation. But what is now particularly referred to, is different from these, as the disease generally makes its approach at a period of life subsequent to that, during which menstruation is continued. This complaint most commonly commences with a sense of weight, and an obtuse pain about the region of the uterus, and by some patients is compared to slight labour pains, which, returning at intervals, and gradually increasing, become a source of great uneasiness.

These symptoms must occasion anxiety in the mind of the medical practitioner, when he considers them indicating disposition in the organ to take on some morbid action, which, too frequently, at a more advanced period, terminates in a schirrous or cancerous state.

The disease is found most commonly amongst those females who have indulged themselves pretty freely in the luxuries of the table. It is attendant particularly upon those who have lived a single life, or who, having been married, have never borne any children; it has generally discovered itself about the time of menstruation ceasing; and it is not improbable, that the means which have been employed to prolong the period of its duration, have contributed, in some degree, to produce the symptoms referred to.

This disease may more easily be prevented, or palliated, than cured. A proper attention to diet and regimen at its first appearance may, for a considerable time, suspend, if not entirely prevent, the occurrence of those more painful and formidable symptoms, which in the progress of the disease, too frequently afflict the patient to such a degree as to render life a burden.

In some cases, where great fullness prevails, the loss of a few ounces of blood may be of essential service; and to keep the bowels regularly open, by the use of some gentle laxative, is indispensably necessary. Every article both of diet and of medicine which is of a heating or stimulating quality must be avoided. Violent exercise may prove injurious, whilst that which is gentle, and not too long continued, will be highly salutary.

